# The Role of Cardiac Biomarkers in the Diagnosis of Hypertensive Emergency

**DOI:** 10.3390/diagnostics13091605

**Published:** 2023-04-30

**Authors:** Mohammed A. Talle, Anton F. Doubell, Pieter-Paul S. Robbertse, Sa’ad Lahri, Philip G. Herbst

**Affiliations:** 1 Division of Cardiology, Department of Medicine, Faculty of Medicine and Health Sciences, Stellenbosch University and Tygerberg Academic Hospital, Cape Town 7505, South Africa; 2 Department of Medicine, Faculty of Clinical Sciences, College of Medical Sciences, University of Maiduguri and University of Maiduguri Teaching Hospital, Maiduguri 600004, Nigeria; 3 Division of Emergency Medicine, Faculty of Medicine and Health Sciences, Stellenbosch University and Tygerberg Academic Hospital, Cape Town 7505, South Africa

**Keywords:** hypertensive emergency, diagnosis, lactate dehydrogenase, high sensitivity cardiac troponin T, brain natriuretic peptide, area under the curve

## Abstract

There is a growing interest in the role of biomarkers in differentiating hypertensive emergency from hypertensive urgency. This study aimed to determine the diagnostic utility of lactate dehydrogenase (LDH), high-sensitivity cardiac troponin T (hscTnT), and N-terminal prohormone of brain-type natriuretic peptide (NT-proBNP) for identifying hypertensive emergency. A diagnosis of hypertensive emergency was made based on a systolic blood pressure of ≥180 mmHg and/or a diastolic blood pressure of ≥110 mmHg with acute hypertension-mediated organ damage. The predictive value of LDH, hscTnT, NT-proBNP, and models of these biomarkers for hypertensive emergency was determined using the area under the receiver operator characteristic curve (AUC). There were 66 patients (66.7% male) with a hypertensive emergency and 16 (31.3% male) with hypertensive urgency. LDH, NT-proBNP, and hscTnT were significantly higher in hypertensive emergency. Serum LDH > 190 U/L and high creatinine were associated with hypertensive emergency. LDH had an AUC ranging from 0.87 to 0.92 for the spectrum of hypertensive emergencies, while hscTnT had an AUC of 0.82 to 0.92, except for neurological emergencies, in which the AUC was 0.72. NT-proBNP was only useful in predicting acute pulmonary edema (AUC of 0.89). A model incorporating LDH with hscTnT had an AUC of 0.92 to 0.97 for the spectrum of hypertensive emergencies. LDH in isolation or combined with hscTnT correctly identified hypertensive emergency in patients presenting with hypertensive crisis. The routine assessment of these biomarkers has the potential to facilitate the timely identification of hypertensive emergencies, especially in patients with subtle and subclinical target organ injury.

## 1. Introduction

Despite improvements in the evaluation and management of systemic hypertension over the last two decades, hypertension and its complications remain a significant public health burden, particularly in low- and middle-income countries [1,2]. Hypertensive crisis with or without evidence of acute hypertension-mediated organ damage remains a common reason for a visit to medical emergency rooms. Patients presenting with symptomatic severe blood pressure elevation (hypertensive crisis) without evidence of acute end organ damage are classified as hypertensive urgency. In contrast, those with evidence of acute hypertension-mediated organ damage are diagnosed as hypertensive emergency [3]. Hypertensive emergency account for about 0.3% of all emergency department patients [4]. Organs affected in patients with hypertensive emergency include the heart, brain, kidney, eyes, and great vessels. However, the most common presentation involves the cardiovascular and neurological systems.

Hypertensive emergency commonly complicates untreated or poorly controlled hypertension; however, it can occur de novo without a prior diagnosis of hypertension. The diagnosis of hypertensive emergency is based on the presenting systolic blood pressure of ≥180 mmHg and/or a diastolic blood pressure of ≥110 mmHg with features of acute hypertension-mediated organ damage [3,5]. The presentation in patients with hypertensive emergency includes acute pulmonary edema, acute coronary syndrome, intracranial hemorrhage, ischemic stroke, hypertensive encephalopathy, aortic dissection, thrombotic microangiopathy, and malignant hypertension [3,4]. In addition to the distinctive clinical features of acute hypertension-mediated organ damage, biomarkers are increasingly used to identify patients with hypertensive emergency [6,7]. However, most hypertension guidelines recommend a context-dependent use of biomarkers (serum and imaging) [3,5]. For instance, lactate dehydrogenase (LDH), a cytoplasmic enzyme found in nearly all body tissues, is only recommended in suspected thrombotic microangiopathy and hemolytic anemia, whereas cardiac troponin assay is limited to patients presenting with features of myocardial ischemia.

In addition to distinct clinical features, there is a growing interest in the role of brain natriuretic peptide (BNP) and other biomarkers in differentiating hypertensive emergency from hypertensive urgency [6,7]. In one study, BNP had an area under the receiver operator characteristic (ROC) curve (AUC) of 0.96 as a diagnostic marker for hypertensive emergency with high sensitivity and specificity among patients presenting with a hypertension crisis [7]. Despite the widespread distribution of LDH and increased levels in patients with hypertensive emergency, its diagnostic utility has not been evaluated beyond diagnosing microangiopathic hemolytic anemia. Our study aimed to determine the diagnostic utility of LDH, high-sensitivity cardiac troponin T (hscTnT), and N-terminal prohormone of BNP (NT-proBNP) for predicting hypertensive emergency in patients presenting with hypertensive crisis.

## 2. Materials and Methods

### 2.1. Study Design and Participants

Patients aged 18 years or older referred to the medical emergency unit of Tygerberg Hospital with hypertensive crises were included in this prospective observational study. Tygerberg hospital is a tertiary hospital in the Western Cape province of South Africa. Patients with hypertensive disorders of pregnancy, patients presenting with ST-elevation myocardial infarction, unconscious patients, and those who declined consent to participate were excluded from the study.

The diagnosis of hypertensive emergency was based on a systolic blood pressure ≥ 180 mmHg or a diastolic blood pressure ≥ 110 mmHg with acute hypertension-mediated organ damage as contained in standard guidelines [3,5,8]. Briefly, a diagnosis of acute pulmonary edema was made based on symptoms/signs of heart failure and chest X-ray findings, while myocardial infarction was diagnosed in the presence of hscTnT levels above 14 ng/L (99th percentile level) with a rising and or falling pattern and features of myocardial ischemia, including abnormalities on electrocardiogram (ECG). A diagnosis of hypertensive encephalopathy was established in the presence of headache, visual disturbances, nausea/vomiting, and grade III–IV retinopathy with or without a seizure, while stroke was diagnosed in the presence of a focal neurological deficit of rapid onset that lasted at least 24 h and confirmed on CT brain. All forms of acute hypertension-mediated organ damage involving the nervous system were grouped and classified as neurological emergencies to form our cohort’s third category of hypertensive emergency.

Routine guideline-directed investigation was carried out in all the patients, including LDH, hscTnT, and NT-proBNP, irrespective of clinical presentation and the type of hypertensive emergency. All blood samples were analyzed at the National Health Laboratory Services laboratory in Tygerberg Hospital (an ISO 15189 accredited laboratory) [9], and normality or otherwise defined based on local reference values. Models of different combinations of the biomarkers were generated based on their predicted probabilities for hypertensive emergencies and their diagnostic utility assessed for the spectrum of hypertensive emergencies.

The human research and ethics committee (HREC) of Stellenbosch University and Tygerberg Academic Hospital approved the study (HREC approval number S19/07/117). All the participants granted consent, and the Declaration of Helsinki was adhered to.

### 2.2. Statistical Analysis

Data was analyzed using SPSS 27 (SPSS Inc, Chicago, IL, USA). The distribution of the data was assessed for normality using the Shapiro–Wilk method. Continuous variables are presented as mean (SD) or median (IQR) and compared using Student’s *t*-test, Mann–Whitney test, and One-way ANOVA as appropriate. Binary regression analysis was used to identify biomarkers associated with a hypertensive emergency. The explanatory variables of interest were assessed individually in a univariate model; those significantly associated with hypertensive emergency were included in a multivariate model to determine their independent contribution. The predictive value of LDH, hscTnT, NT-proBNP, and models of various combinations of these biomarkers was assessed for the composite, and individual subgroups, of hypertensive emergency, using AUC and optimum cut-off point for the biomarkers determined from the individual coordinate of points associated with the ROC curve. The AUC for different biomarkers and models were compared using DeLong test [10]. Results are presented as Tables and Figures. A *p* value of <0.05 was considered significant for all statistical analyses.

## 3. Results

### 3.1. Demographic and Clinical Characteristics

Eighty-two patients with hypertensive crisis were enrolled in the study between May 2021 and November 2022. A total of 66 (80.5%) patients, comprising of 44 (66.7%) males with evidence of acute hypertension-mediated organ damage, were diagnosed as hypertensive emergency. The remaining 16 (19.5%), comprising of 5 (31.3%) males with no evidence of acute target damage, were diagnosed as hypertensive urgency. Age, systolic blood pressure (BP), mean arterial pressure (MAP), and heart rate did not differ significantly between the two groups of hypertensive crises. The clinical and laboratory characteristics of the study participants are illustrated in Table 1.

The most prevalent subtype of hypertensive emergency was acute pulmonary edema (36.4%), followed by neurological emergencies (33.3%) and myocardial infarction (30.3%). Hemoglobin, potassium, sodium, haptoglobin, and platelet count were comparable among the cohort. However, serum creatinine was significantly higher hypertensive emergency (*p* < 0.001). Lactate dehydrogenase (*p* < 0.001), NT-proBNP (*p* = 0.024), and hscTnT (*p* < 0.001) were higher in the patients with hypertensive emergency than hypertensive urgency. Serum LDH was comparable in the subtypes of hypertensive emergency. Patients with acute pulmonary edema had a significantly higher NT-pro BNP (*p* = 0.001) and hscTnT (*p* = 0.010) than patients with neurological emergencies. However, NT-proBNP and hscTnT were similar in patients with acute pulmonary edema and non-ST-elevation myocardial infarction (NSTEMI).

### 3.2. Bivariate and Multivariate Regression Analysis

Using a binary logistic regression, male gender, serum creatinine, estimated glomerular filtration rate (eGFR), serum LDH >190 U/L, serum NT-proBNP > 300 ng/L, and high Cornell voltage indices were significantly associated with a hypertensive emergency. However, only LDH levels > 190 U/L (OR 20, 95%CI 1.66 to 242, *p* = 0.018) and high Cornell voltage indices (OR 1.10, 95%CI 1.00 to 1.21, *p* = 0.045) remained significantly associated with a hypertensive emergency in a multivariate analysis (Table 2). Omnibus tests of model coefficients (likelihood Ration Chi-square 28.3, *p* < 0.001) and the Hosmer and Lemeshow test (*p* = 0.681) confirms fitness of the model, which correctly classified 84.6% of hypertensive emergency. The explanatory variables in the model accounted for 56% (Nagelkerke R square of 0.559) of the occurrence of hypertensive emergency in patients with hypertensive crisis.

### 3.3. Receiver Operator Characteristic

**Figure 1:**
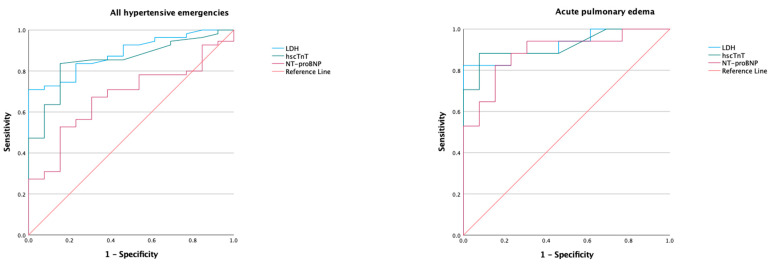

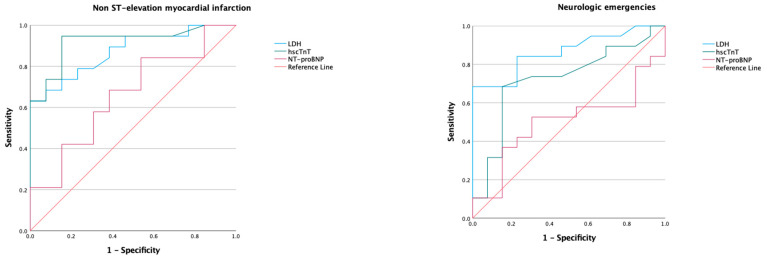
Receiver operating characteristic curve of biomarkers in hypertensive emergency. LDH, lactate dehydrogenase; hscTnT, high-sensitivity cardiac troponin T; NT-proBNP, N-terminal prohormone of brain type natriuretic peptide. Area under the curve (AUC), cut-off points, sensitivity, and specificity are shown in Table 3.

**Figure 2:**
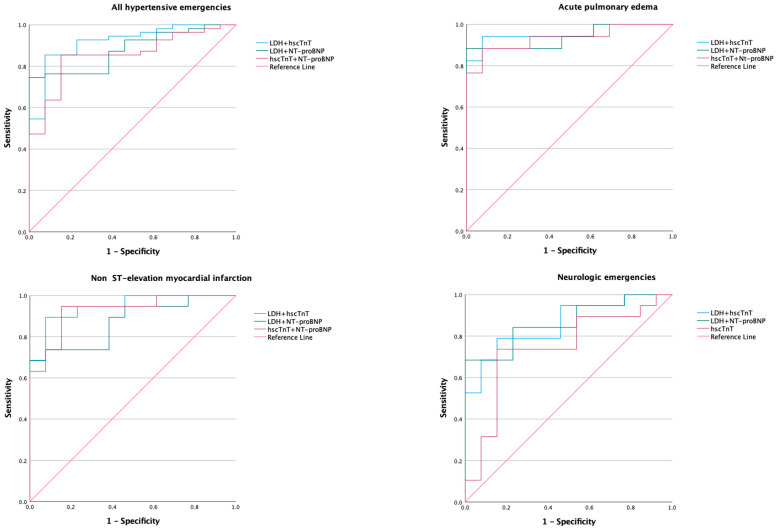
Receiver operating characteristic curve of models of biomarker combinations in hypertensive emergency. LDH, lactate dehydrogenase; hscTnT, high-sensitivity cardiac troponin T; NT-proBNP, N-terminal prohormone of brain type natriuretic peptide. Area under the curve (AUC) are shown in Table 3.

**Figure 3:**
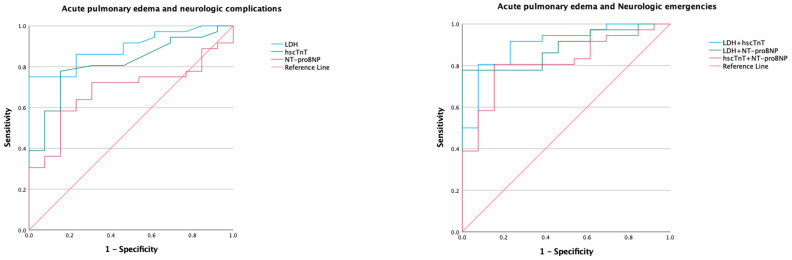
Receiver operating characteristic curve of biomarkers in hypertensive emergency excluding myocardial infarction. LDH, lactate dehydrogenase; hscTnT, high-sensitivity cardiac troponin T; NT-proBNP, N-terminal prohormone of brain type natriuretic peptide. Area under the curve (AUC) are shown in Table 3.

Receiver operator characteristic curves generated for the biomarkers and models of various combinations of biomarkers are presented in Figure 1, Figure 2 and Figure 3; the corresponding AUC, optimum cut-of points, sensitivity, specificity, and likelihood ratios are presented in Table 3.

#### 3.3.1. Lactate Dehydrogenase

Lactated dehydrogenase showed good diagnostic value for the composite of hypertensive emergencies with a moderately increased likelihood at levels above 225 U/L. In the subset with acute pulmonary edema, LDH demonstrated diagnostic value with an AUC of 0.92 and a markedly increased likelihood at levels above 232 U/L. In the subsets with NSTEMI, neurological emergencies, and the composite of acute pulmonary edema and neurological emergencies, LDH maintained a diagnostic value with AUC of 0.87 to 0.89 and a moderately increased likelihood of disease at levels above 218 U/L to 225 U/L. Using the DeLong test, the AUC for LDH did not significantly differ with hscTnT in predicting all forms of hypertensive emergencies (*p* > 0.05 for all). However, LDH had a better AUC for predicting composite of hypertensive emergencies (*p* = 0.004) and neurological emergencies (*p* = 0.001) when compared to NT-proBNP (Table 4).

#### 3.3.2. High-Sensitivity Cardiac Troponin T

High-sensitivity cardiac troponin T had a good diagnostic value for the composite of hypertensive emergencies with an AUC of 0.85 and a markedly increased likelihood at a cut-off level above 14.5 ng/L. In the subgroups with NSTEMI and acute pulmonary edema, the AUC was 0.92, with increased likelihood of these complications at levels above 17.5 ng/L. However, the AUC in the subgroup with neurological emergencies was 0.72, implying a fair diagnostic utility. When compared to NT-proBNP, the AUC for hscTnT was better at discriminating composite of hypertensive emergencies (*p* = 0.023) and NSTEMI (*p* = 0.007).

#### 3.3.3. NT-proBNP

NT-proBNP had a good diagnostic utility with a moderately increased likelihood for the diagnosis of acute pulmonary edema at a cut-off value above 437 ng/L. However, the diagnostic utility was poor for the composite of hypertensive emergencies and NSTEMI, and it failed to differentiate the subgroup with neurological emergencies from hypertensive urgency.

#### 3.3.4. Model of Biomarkers

A model that combined LDH and hscTnT demonstrated good diagnostic utility for the composite of hypertensive emergencies, acute pulmonary edema, and NSTEMI with AUC ranging from 0.92 to 0.96. LDH combined with NT-proBNP showed excellent diagnostic value for acute pulmonary edema and a moderate predictive value for the composite of hypertensive emergency, NSTEMI, and neurological emergencies. However, the AUC for the model of LDH and NT-proBNP is the same as that of LDH.

#### 3.3.5. The Area under the Curve for Biomarkers Based on eGFR

Given the impact of impaired renal function on the biomarkers, we dichotomized the cohort based on eGFR of 60 mL/min/1.73 m^2^. In the subgroup with eGFR > 60 mL/min/1.73 m^2^, LDH and hscTnT had an AUC of 0.83 and 0.76, respectively, for predicting the composite of hypertensive emergency, while for NT-proBNP, the AUC was 0.52. In the subgroup with hypertensive emergency and eGFR < 60 mL/min/1.73 m^2^, LDH and hscTnT had an AUC of 0.94 and 0.97, respectively, while NT-proBNP had an AUC of 0.76.

## 4. Discussion

This study assessed the diagnostic utility of LDH, hscTnT, and NT-proBNP in differentiating hypertensive emergency from hypertensive urgency in patients referred to the Tygerberg hospital with hypertensive crisis. The main findings of our study are as follows:

- LDH consistently showed diagnostic utility in differentiating the spectrum of hypertensive emergency from hypertensive urgency.
- hscTnT demomnstrateddiagnostic utility in the composite of hypertensive emergencies, NSTEMI, and acute pulmonary edema but not in the subgroup with neurological emergencies.
- NT-proBNP has diagnostic value for acute pulmonary edema but has poor utility in the composite of hypertensive emergency and in NSTEMI.
- Models of combinations of LDH and hscTnT had diagnostic utility for the composite of hypertensive emergencies, acute pulmonary edema, and NSTEMI. The combination of LDH with NT-proBNP did not perform differently from LDH.

Our cohort of hypertensive crisis are younger, with comparable age in hypertensive emergency and urgency compared to reports from other studies [11,12,13]. Acute pulmonary edema, neurological emergencies, and acute coronary syndrome dominated the acute hypertension-mediated organ damage, similar to most studies [4,12,13].

To the best of our knowledge, our study is the first to assess the diagnostic utility of LDH in the diagnosis of hypertensive emergency outside of thrombotic microangiopathy and hemolytic anemia. Using the ROC curve, LDH consistently predicted the composite of hypertensive emergency and the different subtypes with AUC ranging from 0.85 to 0.92 and was associated with a moderate to markedly increased likelihood of disease occurrence. Using a model combining LDH with hscTnT resulted in a better diagnostic utility with an AUC of 0.92 to 0.96, except in neurological emergencies where the AUC was 0.86 (Table 3). Lactate dehydrogenase was previously used to diagnose myocardial infarction; however, its role in this regard has become historical with the advent of cardiac troponin [14]. The main indication for serum LDH assays in patients with hypertensive emergency is for the diagnosis of hemolytic anemia resulting from thrombotic microangiopathy [3]. Despite the lack of other evidence for hemolytic anemia, LDH was significantly higher in all subtypes of hypertensive emergency than in the group with hypertensive urgency and predicted all forms of hypertensive emergency.

Lactate dehydrogenase is ubiquitously distributed in all body tissues, and its levels can increase in conditions including anemia, muscle trauma, myocardial infarction, kidney injury, and infections, among others [15]. Given the uneven distribution of the five isoenzymes of LDH (LD1 isoenzyme predominantly in the heart, red blood cell, and kidney, and LD5 is mainly located in the liver and skeletal muscle), it is plausible that assaying specific isoenzymes can improve its specificity in identifying organ injury in patients with hypertensive emergency, including subclinical acute hypertension-mediated organ injury. The predictive role of LDH in patients with hypertensive emergency (composite and subtypes) demonstrated in this study and its prognostic roles established in other studies [16,17] warrants a larger study to determine the role of its routine assessment in patients presenting with hypertensive crisis, as this will assist with the timely identification of hypertensive emergency, including subtle and subclinical cases. Furthermore, LDH may be used to risk stratify and prognosticate patients with hypertensive emergency.

The guidelines on evaluating and managing hypertensive emergency recommend cardiac troponin assay only when myocardial infarction is suspected [3,5]. An acute myocardial injury without features of myocardial ischemia can complicate hypertensive emergency [18]. However, the diagnostic value of cardiac troponin for hypertensive emergency has not been studied outside myocardial infarction. High-sensitivity cardiac troponin T demonstrated good diagnostic utility for the composite of hypertensive emergency. As expected, hscTnT performed excellently in predicting NSTEMI and acute pulmonary edema but only performed fairly for neurological emergencies. The comparable diagnostic performance in acute pulmonary edema and NSTEMI reflects the high rate of myocardial injury in patients presenting with hypertensive emergency and acute pulmonary edema. However, it will be difficult to exclude overlap between acute pulmonary edema and NSTEMI, given their shared and non-specific clinical presentation and ECG findings. The better prediction for NSTEMI and acute pulmonary edema provided by a model of combined LDH and hscTnT is probably due to the complementary ability of these two biomarkers to detect cardiac injury. On the other hand, the poor sensitivity of hscTnT for neurological emergencies when compared to acute pulmonary edema may be related to the low rate of myocardial injury in the former. In addition to its role in diagnosing myocardial injury, risk stratification, and prognosis [18], the predictive value of hscTnT for hypertensive emergency, irrespective of myocardial infarction, supports its routine use in the evaluation of patients with hypertensive crisis.

In contrast to the lack of literature on the diagnostic value of LDH for hypertensive emergency in general, some studies have assessed NT-proBNP in this group, including its diagnostic utility and prognostic implications [6,7,19]. In one study involving thirty patients with hypertensive crisis (equal number of hypertensive emergency and urgency), BNP was reported to be an excellent diagnostic marker for hypertensive emergency (AUC of 0.96) with high sensitivity and specificity [7]. Their report contrasts our finding of an AUC of 0.62 (*p* = 0.059) for the composite of hypertensive emergency. Although NT-proBNP had an AUC of 0.89 in our patients with acute pulmonary edema, the study that reported an AUC of 0.96 did not include acute pulmonary edema or acute heart failure [7], and they excluded patients with eGFR < 60 mL/min/1.73 m^2^, a group that constituted nearly half of our patients with hypertensive emergency. However, a subgroup analysis of our cohort with eGFR > 60 mL/min/1.73 m^2^ revealed an AUC of 0.52 for NT-proBNP. Second, 27% of our patients with hypertensive urgency had NT-proBNP > 300 ng/L, and this might have impacted its diagnostic utility in differentiating hypertensive emergency from hypertensive urgency in our cohort. The good diagnostic utility of NT-proBNP for differentiating acute pulmonary edema from hypertensive urgency in our study is not unexpected, given the established diagnostic and prognostic roles of the biomarker in all forms of heart failure [20,21,22]. Increased wall stress, the most common stimulus for BNP release from the cardiac myocytes, occurs in patients with acute pulmonary edema and increased left ventricular filling pressure [23,24]. In this regard, it is intuitive and reasonable that a model of LDH combined with NT-proBNP yielded a better AUC of 0.94.

Lactate dehydrogenase, hscTnT and NT-proBNP share in common the attribute of being either a direct or surrogate indicator of acute hypertension-mediated organ damage that define hypertensive emergency. Our study showed higher levels of these biomarkers in patients with hypertensive emergency compared to hypertensive urgency. Given the common pathophysiologic mechanism underpinning hypertensive emergency, multiple hypertension-mediated organ damage (including subclinical organ injury) could occur to result in concurrent elevation of these biomarkers, and partly explains their shared diagnostic performance for the composite of hypertensive emergency and the subtypes, particularly, LDH and hscTnT. A larger study is required to define the future role of routine assessment of these biomarkers in identifying hypertensive emergency among patients presenting with hypertensive crisis.

### Limitation of the Study

Our study has a number of limitations. Being a single-center study with a small sample size, it might not be sufficiently powered to differentiate hypertensive emergency from hypertensive urgency, resulting in a type-2 error. However, the study provides an important proof-of-concept for the diagnostic utility of LDH, hscTnT, and NT-proBNP in differentiating hypertensive emergency from hypertensive urgency. Second, almost 50% of the patients with hypertensive emergency had an eGFR < 60 mL/min/1.73 m^2^, which might have impacted the biomarkers, especially hscTnT and NT-proBNP. However, LDH maintained an AUC of 0.94 and 0.83 in hypertensive emergency patients with and without renal impairment, respectively. Third, optimum cut-off values for the model of biomarkers could not be determined because the models were generated based on probabilities. Fourth, aortic dissection, thrombotic microangiopathy, and pre-eclampsia were not represented in our cohort, and therefore, the findings may not apply to the whole spectrum of hypertensive crisis. Finally, the training and testing of the ROC model was not completed due to the small sample size.

## 5. Conclusions

Using AUC, we have demonstrated the potential diagnostic utility of LDH, hscTnT, and NT-proBNP in differentiating hypertensive emergency from hypertensive urgency in patients presenting with hypertensive crisis. Our data also illustrated the diagnostic performance of models using a combination of these biomarkers in identifying hypertensive emergency. A larger study is required to determine the role of the routine assessment of these and other novel biomarkers in facilitating timely identification of hypertensive emergency, including subtle and subclinical target organ injuries.

## Figures and Tables

**Table 1:** Comparison of clinical and laboratory characteristics in hypertensive urgency and hypertensive emergency.

Variables	Hypertensive Crisis		*p*-Value	Type of Hypertensive Emergency			*p*-Value *
	Hypertensive Urgency (*n* = 16)	Hypertensive Emergency (*n* = 66)		Acute Pulmonary Edema (*n* = 24)	Non-ST Elevation Myocardial Infarction (*n* = 20)	Neurological Emergencies (*n* = 22)	
Age in years, mean (SD)	49.5 (15.6)	47.9 (13.2)	0.673	48.0 (15.7)	53.2 (11.6)	42.8 (9.6)	0.099
Males, *n* (%)	5 (31.3)	44 (66.7)	0.077	15 (62.5)	14 (70)	15 (68.2)	
Systolic blood pressure (mmHg)	216.1 (27.6)	217.1 (28.8)	0.902	223.4 (27.3)	202.9 (26.7)	223.2 (29)	0.094
Diastolic blood pressure (mmHg)	118.4 (16.6) ^b,d^	130.2 (19.9)	0.015	136 (18.5) ^a,c^	116.7 (16.8) ^b,d^	136.1 (18.5) ^a,c^	<0.001
Mean arterial pressure (mmHg)	151 (16.8)	159.2 (20.4)	0.122	165.1 (18.6) ^c^	145.4 (17.2) ^b,d^	165.2 (19.4) ^c^	<0.001
Cornell voltage indices (mm)	21.5 [5.8–28.3] ^b^	30 [21–40]	0.005	37 [30.3–45.8] ^a,c^	21.5 [17.5–26.5] ^b^	27 [18.5–43]	<0.001
Hemoglobin (g/dL)	13.8 (1.8)	13.8 (2.2)	0.810	13 (2.0)	14.3 (2.3)	14.1 (2)	0.155
Platelet count (×10^9^/L)	276.5 [250.5–373]	283 [252–346]	0.951	286 [254.3–343.8]	286 [268–440]	277 (202.5–340.5)	0.803
Creatinine (μmol/L)	82.5 [64–99.5] ^b^	110.5 [90.8–190.8]	<0.001	192.5 [101–266]	107.5 [90.3–124]	103.5 (82–119)	<0.001
eGFR (mL/min/1.73 m^2^	81.5 [64–105.8] ^b^	63 [36.5–79.5]	0.006	36.5 [23–65.5] ^a,d^	66.5 [43.3–80.5]	71.5 [62.8–98.5] ^b^	<0.001
Lactate dehydrogenase (U/L)	200 [170–231] ^b,c,d^	283 [230.8–330.5]	<0.001	282 [269.5–342.5] ^a^	285 [226–353] ^a^	282 [229–314.3] ^a^	<0.001
Haptoglobin (g/L)	1.93 [1.6–2.3]	1.97 [1.22–2.64]	0.797	1.88 [1.08–2.68]	2.3 [1.72–2.87]	1.97 [1.1–2.6]	0.600
hscTnT (ng/L)	11 [8–14] ^b,c^	40 [17.5–165]	<0.001	56.5 [32.3–161.5] ^a,d^	182 [25–277.3] ^a,d^	17 [12–25] ^b,c^	<0.001
NT-proBNP (ng/L)	136 [51–396] ^b^	528 [113–2117]	0.024	1985 [532.8–4577] ^a,d^	315 [113–1435]	151 [21.5–845.5] ^b^	<0.001

* *p*-value for One-way ANOVA comparing types of hypertensive emergency and hypertensive urgency. eGFR; estimated glomerular filtration rate; hscTnT, high-sensitivity cardiac troponin T; NT-proBNP, N-terminal prohormone of brain natriuretic peptide. ^a^ *p* < 0.05 versus hypertensive urgency. ^b^ *p* < 0.05 versus acute pulmonary edema. ^c^ *p* < 0.05 versus non-ST elevation myocardial infarction. ^d^ *p* < 0.05 versus neurological emergencies.

**Table 2:** Predictors of hypertensive emergency in patients with hypertensive crisis.

Variable	Unadjusted OR (95% CI)	*p*-Value	Adjusted OR (95% CI)	*p*-Value *
Age in years, mean (SD)	0.99 (0.95–1.04)	0.685	-	-
Male gender	0.23 (0.07–0.74)	0.013	4.57 (0.69–30.4)	0.116
Systolic BP (mmHg)	1.00 (0.98–1.02)	0.898	-	-
Diastolic BP (mmHg)	1.04 (1.00–1.07)	0.047	**-**	**-**
Mean arterial pressure (mmHg)	1.02 (0.99–1.05)	0.115	-	-
Creatinine (μmol/L)	1.04 (1.01–1.06)	0.004	1.03 (0.99–1.08)	0.111
Hemoglobin (g/dL)	0.99 (0.79–1.26)	0.976	-	-
LDH > 190 U/L	14.7 (3.53–62.4)	<0.001	20.04 (1.66–242)	0.018
Haptoglobin (g/L)	1.23 (0.85–1.79)	0.271	-	-
Haptoglobin > 2.0 (g/L)	1.51 (0.49–4.67)	0.474	-	-
Platelet × 10^9^/L	1.00 (0.99–1.00)	0.860	-	-
hscTnT (ng/L)	1.10 (0.97–1.25)	0.121	-	-
NT-proBNP (ng/L)	1.00 (1.00–1.01)	0.107	-	-
NT-proBNP > 300 ng/L	4.02 (1.15–14.0)	0.029	0.20 (0.02–2.06)	0.175
Cornell voltage indices (mm)	1.09 (1.032–1.15)	0.002	1.10 (1.00–1.21)	0.045

* p value for multivariate analysis. hscTnT, high sensitivity cardiac troponin T; LDH, lactate dehydrogenase; NT-proBNP, N-terminal prohormone of brain-type natriuretic peptide. Hypertensive urgency was used as a reference group for the analysis.

**Table 3:** Potential cut off levels of biomarkers for predicting hypertensive emergency.

Type of Hypertensive Emergency and Biomarker	AUC (95% CI)	Cut-Off Point *	Sensitivity, %	Specificity, %	Positive Likelihood Ratio	Negative Likelihood Ratio	*p* Value for AUC
**All hypertensive emergencies:**							
LDH	0.88 (0.80 to 0.97)	225	83	77	3.11	0.22	<0.001
hscTnT	0.85 (0.80 to 0.96)	14.5	83	85	5.60	0.19	<0.001
NT-proBNP	0.67 (0.53 to 0.81)	208	67	69	2.06	0.52	0.059
LDH + hscTnT	0.92 (0.84 to 1.00)	-	-	-			<0.001
LDH + NT-proBNP	0.88 (0.80 to 0.96)	-	-	-			<0.001
hscTnT + NT-proBNP	0.85 (0.75 to 0.95)	-	-	-			<0.001
Acute pulmonary edema:							
LDH	0.92 (0.83 to 1.00)	232	82	85	5.47	0.21	<0.001
hscTnT	0.92 (0.82 to 1.00)	17.5	88	85	5.87	0.14	<0.001
NT-proBNP	0.89 (0.77 to 1.00)	437	88	77	3.83	0.16	<0.001
LDH + hscTnT	0.96 (0.88 to 1.00)	-	-	-			<0.001
LDH + NT-proBNP	0.94 (0.85 to 1.00)	-	-	-			<0.001
hscTnT + NT-proBNP	0.93 (0.84 to 1.00)	-	-	-			<0.001
**Non-ST-elevation myocardial infarction:**							
LDH	0.87 (0.75 to 0.99)	218	84	61	2.15	0.16	<0.001
hscTnT	0.92 (0.82 to 1.00)	17.5	90	85	6.00	0.12	<0.001
NT-proBNP	0.66 (0.47 to 0.085)	202	58	62	1.53	0.68	0.130
LDH + hscTnT	0.95 (0.88 to 1.00)	-	-	-			<0.001
LDH + NT-proBNP	0.87 (0.75 to 0.99)	-	-	-			<0.001
hscTnT + NT-proBNP	0.93 (0.84 to 1.00)	-	-	-			<0.001
**Neurological emergencies:**							
LDH	0.87 (0.74 to 0.99)	225	84	77	3.65	0.21	0.001
hscTnT	0.72 (0.54 to 0.91)	14.5	68	85	4.53	0.38	0.035
NT-proBNP	0.50 (0.30 to 0.71)	208	53	70	1.77	0.67	0.985
LDH + hscTnT	0.86 (0.73 to 0.99)	-	-	-			0.001
LDH + NT-proBNP	0.87 (0.74 to 0.99)	-	-	-			0.022
hscTnT + NT-proBNP	0.74 (0.56 to 0.92)	-	-	-			0.001
**Acute pulmonary edema and neurological emergencies:**							
LDH	0.89 (0.81 to 0.98)	225	86	77	3.74	0.18	<0.001
hscTnT	0.82 (0.69 to 0.94)	18.5	78	85	5.20	0.26	0.001
NT-proBNP	0.68 (0.53 to 0.84)	215	69	70	2.30	0.44	0.051
LDH + hscTnT	0.91 (0.82 to 0.99)	-	-	-			<0.001
LDH + NT-proBNP	0.89 (0.79 to 0.98)	-	-	-			0.001
hscTnT + NT-proBNP	0.81 (0.69 to 0.94)	-	-	-			<0.001

AUC, area under the curve; LDH, lactate dehydrogenase; hscTnT, high sensitivity cardiac troponin T; NT-proBNP, N-terminal prohormone of brain natriuretic peptide. * LDH in U/L, hscTnT and NT-proBNP measured in ng/L. Cut-off points, sensitivity, and specificity are not provided for models because they are generated based on probabilities.

**Table 4:** Pairwise comparison of AUC using DeLong test.

Biomarker and Models	Difference between Areas	Standard Error	95% CI	z Statistic	*p* Value
LDH vs. hscTnT	0.035	0.057	−0.077 to 0.147	0.611	0.541
LDH vs. NT-proBNP	0.210	0.072	0.067 to 0.352	2.912	0.004
LDH vs. LDH+hscTnT	0.033	0.030	−0.026 to 0.092	1.094	0.274
LDH vs. LDH+NT-proBNP	0.006	0.012	−0.018 to 0.031	0.510	0.610
LDH vs. hscTnT+NT-proBNP	0.033	0.058	−0.082 to 0.147	0.563	0.573
hscTnT vs. NT-proBNP	0.176	0.077	0.024 to 0.327	2.277	0.023
hscTnT vs. LDH+hscTnT	0.068	0.037	−0.004 to 0.139	1.855	0.064
hscTnT vs. LDH+NT-proBNP	0.029	0.058	−0.086 to 0.143	0.492	0.623
hscTnT vs. hscTnT+NT-proBNP	0.002	0.005	−0.008 to 0.012	0.398	0.690
NT-proBNP vs. LDH+hscTnT	0.243	0.074	0.099 to 0.388	3.306	<0.001
NT-proBNP vs. LDH+NT-proBNP	0.204	0.065	0.076 to 0.332	3.124	0.002
NT-proBNP vs. hscTnT+NT-proBNP	0.178	0.079	0.022 to 0.333	2.242	0.025
LDH+hscTnT vs. LDH+NT-proBNP	0.039	0.034	−0.027 to 0.105	1.168	0.243
LDH+hscTnT vs. hscTnT+NT-proBNP	0.066	0.038	−0.008 to 0.140	1.741	0.082
LDH+NT-proBNP vs. hscTnT+NT-proBNP	0.027	0.060	−0.090 to 0.144	0.445	0.656

LDH, lactate dehydrogenase; hscTnT, high-sensitivity cardiac troponin T; NT-proBNP, N-terminal prohormone of brain-type natriuretic peptide; CI, confidence interval.

## Data Availability

The authors declare their willingness to share the data for this research upon a reasonable request, and approval by the Stellenbosch University.
